# Development and verification of a 7-lncRNA prognostic model based on tumor immunity for patients with ovarian cancer

**DOI:** 10.1186/s13048-023-01099-0

**Published:** 2023-02-04

**Authors:** Jing Feng, Yiping Yu, Wen Yin, Sumin Qian

**Affiliations:** Gynecology Department 2, Cangzhou Central Hospital, No. 16, Xinhua West Road, Yunhe District, Cangzhou, Hebei Province 061000 China

**Keywords:** Ovarian cancer, ssGSEA, lncRNA, Prognostic signature, Rapamycin

## Abstract

**Background:**

Both immune-reaction and lncRNAs play significant roles in the proliferation, invasion, and metastasis of ovarian cancer (OC). In this study, we aimed to construct an immune-related lncRNA risk model for patients with OC.

**Method:**

Single sample GSEA (ssGSEA) algorithm was used to analyze the proportion of immune cells in The Cancer Genome Atlas (TCGA) and the hclust algorithm was used to conduct immune typing according to the proportion of immune cells for OC patients. The stromal and immune scores were computed utilizing the ESTIMATE algorithm. Weighted gene co-expression network analysis (WGCNA) and differentially expressed genes (DEGs) analyses were utilized to detect immune cluster-related lncRNAs. The least absolute shrinkage and selection operator (LASSO) regression was conducted for lncRNA selection. The selected lncRNAs were used to construct a prognosis-related risk model, which was then validated in Gene Expression Omnibus (GEO) database and in vitro validation.

**Results:**

We identify two subtypes based on the ssGSEA analysis, high immunity cluster (immunity_H) and low immunity cluster (immunity_L). The proportion of patients in immunity_H cluster was significantly higher than that in immunity_L cluster. The ESTIMATE related scores are relative high in immunity_H group. Through WGCNA and LASSO analyses, we identified 141 immune cluster-related lncRNAs and found that these genes were mainly enriched in autophagy. A signature consisting of 7 lncRNAs, including AL391832.3, LINC00892, LINC02207, LINC02416, PSMB8.AS1, AC078788.1 and AC104971.3, were selected as the basis for classifying patients into high- and low-risk groups. Survival analysis and area under the ROC curve (AUC) of the signature pointed out that this risk model had high accuracy in predicting the prognosis of patients with OC. We also conducted the drug sensitive prediction and found that rapamycin outperformed in patient with high risk score. In vitro experiments also confirmed our prediction.

**Conclusions:**

We identified 7 immune-related prognostic lncRNAs that effectively predicted survival in OC patients. These findings may offer a valuable indicator for clinical stratification management and personalized therapeutic options for these patients.

**Supplementary Information:**

The online version contains supplementary material available at 10.1186/s13048-023-01099-0.

## Introduction

Ovarian cancer (OC) is one of the most lethal cancers with high mortality. By 2020, more than 300,000 new cases of OC are expected to occur worldwide, accounting for 3.6% of all cancer diagnoses, with more than 190,000 deaths expected [1]. Moreover, due to its insidious clinical presentation and no effective screening method in the early stage, most cases (almost 75%) are diagnosed at late stage, resulting in a poor 5-year survival rate [2]. Despite advances in combination chemotherapy, targeted therapy, and intraperitoneal chemotherapy, 80% of OC patients initially respond to treatment, chemotherapy resistance followed by recurrent disease remains common in OC [3]. Therefore, early diagnosis and treatment are crucial to improve the quality of life and survival rate of OC patients. The advances have demonstrated that OC with sufficient heterogeneity contributes to treatment failure and a poor prognosis [4]. Consequently, to explore and establish a reliable prognostic model of OC is an urgent problem to be solved to guide more appropriate clinical treatment and improve the prognosis of OC.

Long noncoding RNAs (lncRNAs) are a family of nonprotein-coding RNAs longer than 200 nucleotide [5]. Recent studies have demonstrated that abnormal expression of various lncRNAs has been detected to play key roles in tumorigenesis and progression [6, 7]. The presence of lncRNAs is closely related to the recurrence, metastasis and prognosis of OC, suggesting that lncRNAs can be used as new potential molecular markers for tumor prognosis. Zheng et al. reported that lncRNAs involved in m6A regulation (LI-m6As) can independently predict the OS and therapeutic value of OC [8]. However, lncRNAs involved in the immune response of OC remain unclear.

Although the genetic and epigenetic changes in tumor cells are crucial to the oncogenesis and progress of tumors, accumulating evidence shows that the interaction between tumor cells and its surrounding normal cells also plays an important role [9]. The tumor microenvironment (TME) is a heterogeneous system composed of cancer cells, extracellular matrix, immune cells, as well as other molecules [10]. As the major cellular components of the TME, the immune infiltrating cells and stromal cells are getting more and more attention. Evaluation of the status of these two types of cells in TME will contribute to more accurate diagnosis and prognosis evaluation of tumor patients. Immunity is an important part of TME. Therefore, understanding the immune-related characteristics of OC is of great significance for its risk stratification and targeted therapy [11]. The Estimation of Stromal and Immune cells in Malignant Tumor tissues using Expression data (ESTIMATE) method has been successfully applied to the quantitative analysis of TME of various tumors, and its effectiveness has been proved. Up to now, although many studies have analyzed OC patients from the perspective of immune cell infiltration [12], there is a lack of joint exploration of the relationship between OC and immunity from many immune aspects, such as immune-related genes, immune cell infiltration and transcription factors (TFs).

In our study, we downloaded the expression profile of OC patients from the TCGA database and divided tumor samples from TCGA-OC into high immunity (immunity_H) and low immunity (immunity_L) group through single sample gene set enrichment analysis (ssGSEA). Then, we further revealed the key lncRNA that played important roles in this immune group by WGCNA and successfully classified OC patients into two subtypes. Last but not least, we constructed a nomogram that would be convenient for clinicians to judge the prognosis of OC patients. This risk model was externally validated with GEO database. Immune-related lncRNAs may be potential biomarkers and provide new ideas for immunotherapy.

## Methods

### Data collection

The RNA-sequence profiles and corresponding clinical data of 379 patients with ovarian cancer were downloaded from TCGA (https://portal.gdc.cancer.gov/) and GEO (https://www.ncbi.nlm.nih.gov/geo/) (GSE17260, *n* = 110; GSE14764, *n* = 55). Meanwhile, the corresponding clinical information of OC patients, including patient age, grade, stage, status and histological type of tumor, were also downloaded. We processed the related data of TCGA and GEO datasets through perl (strawberry-perl-5.30.0.1-64b it) and R software, making the data easy to understand and visualize. The lncRNAs were set as up-regulated-lncRNAs with logFC > 1 and *P*-value < 0.05 and down-regulated-lncRNAs with with logFC <-1 and *P*-value < 0.05. Both the DEGs obtained were then analyzed for expression differences.

### Implementation of single‐sample gene set enrichment analysis (ssGSEA)

Single Sample Gene Sets Enrichment Analysis (ssGSEA) was performed on TCGA-OC samples based on signature genes represented by 29 immune cells or immune-related functions using R packages (“GSVA”, “limma”, “GSEABase”) [13]. According to the immune characteristics of 379 TCGA-OC samples, the samples were divided into two subtypes, including the high immunity group (Immunity_H) and the low immunity group (Immunity_L) by using “hclust” (R package) [14]. The bio-similarity of tumor-infiltrating immune cells was estimated using the multidimensional scaling and Gaussian fitting model.

### Verification of the effectiveness of immune grouping

The Stromal Score, Immune Score, ESTIMATE Score, and Tumor Purity were also analyzed by ESTIMATE algorithm based on transcriptome expression profiles of ovarian cancer to verify the effect of ssGSEA grouping and to draw clustering heatmap and statistical map [15]. The gene expression level of human leukocyte antigen (HLA) were used to verify the differences between the two groups. The CIBERSORT deconvolution algorithm was used to accurately determine the composition of immune cells in large tumor sample data from mixed cell types, and the DEGs of the two groups was verified again.

### WGNCA for the transcriptome of ovarian cancer

WGCNA was used to recognize the relationship between co-expressed lncRNA modules and immune cluster. Module eigengenes (MEs) were defined as the first principal component of each lncRNA module and adopted as the representative of all lncRNA in each module. Gene significance (GS), as the mediator p-value for each gene, represented the degree of linear correlation between gene expression of the module and clinical features. Cluster related modules were defined as *P* ≤ 0.01 and the higher GS value was extracted for further analysis.

### Comparative analysis of GO and KEGG pathways in key module

The Kyoto Encyclopedia of Genes and Genomes (KEGG) and Gene Ontology (GO) functional analyses were used to examine the functions of key lncRNA in the module from WGCNA. The lncRNAs were grouped into three categories derived from the findings of the GO analysis: biological processes (BP), molecular functions (MF), and cellular components (CC). The R packages “limma,” “org.Hs.eg.db,” “dose,” “clusterprofiler,” and “enrichplot” were employed. It was determined which pathways were active in the high- and low-risk groups using lncRNA set enrichment analysis (GSEA). There was statistical significance when | NES |≥ 1 and FDR q < 0.05 were used.

### Construction of the module related risk signature

We first performed unsupervised clustering analysis with the “ConsensusClusterPlus” package. The samples were classified into different subtypes based on the expression of key lncRNA. LASSO is a regularization and descending dimension method which can be used in biomarker screening for survival analysis combined with the Cox model [16]. To further evaluate the prognostic impact of these key lncRNAs in WGCNA module and their significance in survival status, we employed Cox regression analysis with the cut-off *P*-value of 0.01. Prognosis-related DEGs were then extracted and employed for the subsequent the LASSO analysis, which could narrow down the candidate lncRNA with the minimum criteria of penalty parameter (λ) and further generate a multiple-lncRNA signature. After centralizing and standardizing the expression value of TCGA-OC cohort, we calculated the risk score of each sample and obtained its formula = coefficient lncRNA1 expression of lncRNA1 + coefficient lncRNA2 expression of lncRNA2 + … + coefficient lncRNAs expression of lncRNAn. According to the median risk score, we separated OC samples into 2 groups (high- and low-risk) and performed Kaplan–Meier analysis to compare overall survival (OS) between different risk groups. The “timeROC”, “survival”, “survminer”, and “survivalROC” packages were used to perform 1-, 3-, 5-year receiver operating characteristic (ROC) analysis of this prognostic signature and compared the area under the ROC curve (AUC) of this signature and other clinicopathological traits.

### Immune infiltration analysis

Analyzing the infiltration of immune cells in cancer has a crucial guiding role in disease research and treatment prognosis prediction. CIBERSORT is an algorithm for deconvolution of the expression matrix of immune cell subtypes based on the principle of linear support vector regression, and the LM22 eigengene matrix can be used to predict the degree of 22 kinds immune cells infiltration in all samples of a dataset [17]. We also used the CIBERSORT algorithm to evaluate the abundance of 22 kinds immune cell species and analyze the relationship between hub- lncRNA and different immune cells based on the dataset.

### Construction of the comprehensive predictive model

To evaluate whether this signature was an independently predictive factor for OC prognosis, we entered this signature and clinicopathological features into the univariate and multivariate Cox regression analysis. Moreover, by integrating of this signature and clinicopathological characteristics, we constructed a quantitative method by which clinicians could predict OC patients’ OS.

### Prediction of the half-maximal inhibitory concentration (IC50) for different risk groups

The effect of chemotherapy was predicted by R package “pRRophetic” [18], which was based on a ridge regression model to calculate the half-maximal IC50 of drugs.

### Wound healing and transwell assays

Cell migration was detected by wound healing and transwell assays. Cells were seeded into 6-well plates and cultured until the confluence reached 95%. A sterile 10 μl pipette tip was used to generate a scratch through each well. The wound closure was observed after 0 h and 24 h and photographed under a microscope (Olympus, Tokyo, Japan). For cell migration assays, ovarian cancer cell lines (SKOV3 and A2780) were added to the upper chambers in Dulbecco’s modified eagle medium (DMEM) containing 1% fetal bovine serum (FBS). The lower chambers were filled with DMEM containing 20% FBS. After a 24-h incubation, the upper chambers were fixed with methanol at room temperature for 30 min and stained with crystal violet staining solution for an additional 30 min. The cells that passed through the membrane were counted under a Leica microscope (magnification, × 100).

### MTT assay

Cell (1 × 10^5^) viability was determined by MTT Kit (Beyotime, Shanghai, China). The cells were seeded in 96-well plates and incubated for 24, 48, or 72 h (h). 10 μL MTT was added with for 4 h. Then, 100 μL DMSO was added to each well and incubated with for 2 h. The optical density (OD) value was measured at 490 nm wavelength and each experiment was repeated for three times.

### Animal model of tumor xenograft

Four-week-old female BALB/C nude mice were purchased from the Charles River Company. All experiments were performed in accordance with the official recommendations of the Chinese animal community. Ovarian cancer cell lines SKOV3 cell lines were established in nude mice. The suspension of the two groups, containing 2 × 10^6^ cells, was injected into the abdominal cavity (5 mice for each group). On the 30th day after intraperitoneal injection, mice were sacrificed by cervical decapitation, and mice models died before being sacrificed were excluded. Peritoneal spreading and metastatic tumor numbers were then counted and photographed.

### Statistical analysis

All data calculations and statistical analyses were performed using R programming (https://www.r-project.org/, version 4.0.2). For comparison two groups of continuous variables, independent Student *t*-tests were used to calculate the differences between normally distributed variables, and Mann–Whitney U-tests were used to calculate the differences between non-normally distributed variables. ROC curves were plotted using tdROC package, and the AUC was counted to estimate the accuracy of risk score in prognosis. All the bilateral statistical *P* values were statistically significant at *P* < 0.05.

## Results

### Identification and preliminary evaluation of two subtypes of OC

**Fig. 1:**
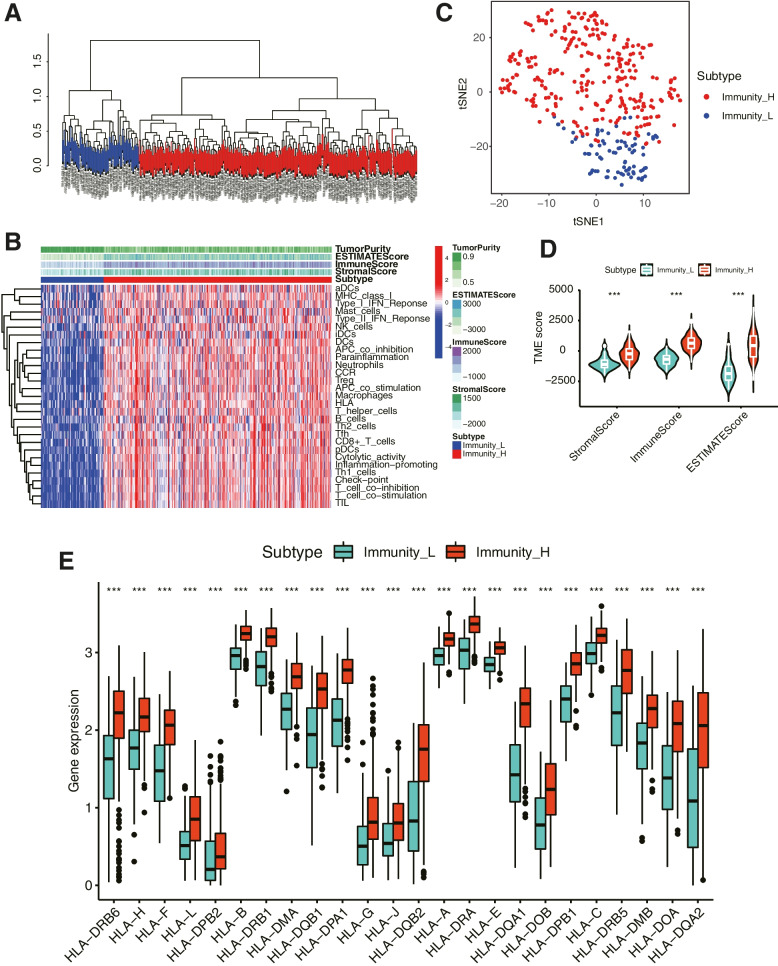
Immune subtypes and clustering in OC patients. **A** Based on the results of ssGSEA, OC patients were divided into Immunity_H and Immunity_L by hierarchical clustering algorithm. **B** Immune infiltration and tumor microenvironment landscape of TCGA-OC patients. **C** Verification of immune subtypes by tSNE. **D** The comparison of Immune Score, Stromal Score and ESTIMATE Score in Immunity_H and Immunity_L groups. **E** Comparison of the expression levels of HLA genes between two subtypes

According to the immunological characteristics of 379 tumor samples in the TCGA-OC cohort, we divided them into high immunity group (Immunity_H) and low immunity group (Immunity_L) based on 29 immune gene sets along with ssGSEA algorithm. R packages (“estimate”, “limma”) were used to calculate the Immune score, Stromal score, ESTIMATE score and Tumor Purity of the two subtypes (Fig. 1A). The heatmap of immune responses based on the ESTIMATE algorithms and single-sample GSEA (ssGSEA) is depicted in Fig. 1B. We further used tSNE algorithm for clustering analysis of TCGA-OC and obtained similar classification results (Fig. 1C). In addition, the results revealed the Immune Score, Stromal Score, and ESTIMATE Score of the Immunity_H was higher than that of the Immunity_L. Moreover, the violin plot also showed significant differences in Immune Score, Stromal Score and ESTIMATE Score between the two subtypes (Fig. 1D). We further explored the expressions of HLA genes between the two subtypes and discovered that the expressions of all HLA genes in Immunity_H were significantly higher than that in Immunity_L (Fig. 1E). These results illustrated the significance of our classification of OC into two subtypes, which could largely distinguish the characteristics of OC.

### Detection of immunity‐related module and hub genes by WGANA

**Fig. 2:**
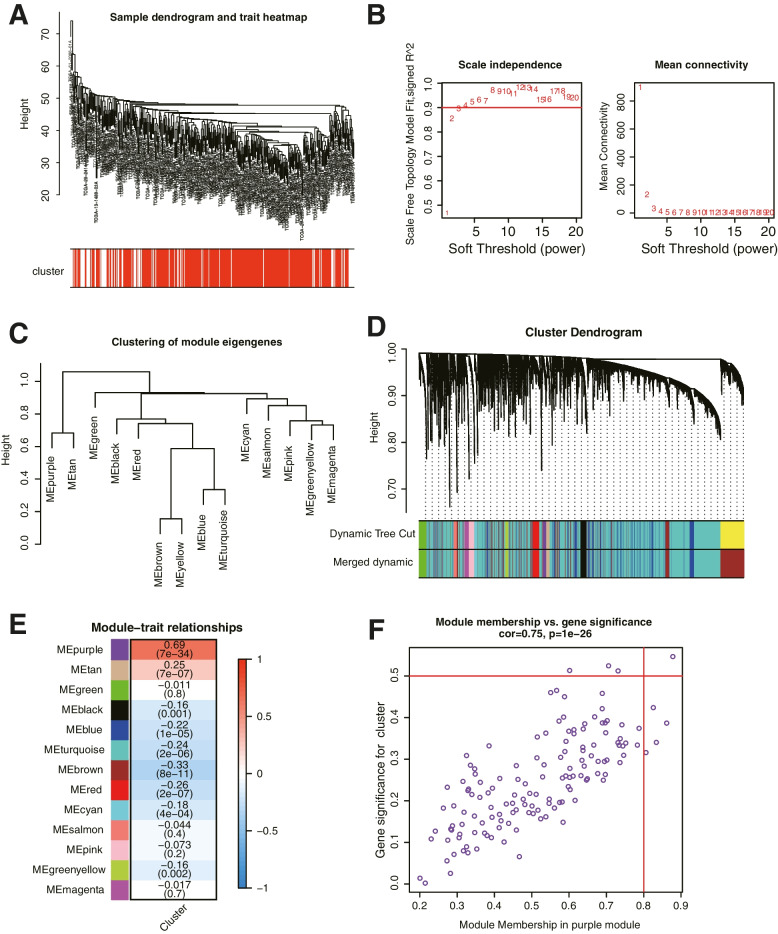
Detection and validation of immunity-related module by WGCNA. **A** The cluster was based on the transcriptome data from TCGA. The color intensity represents the immunity cluster. **B** Analysis of the scale-free fit index for various soft-thresholding powers and the mean connectivity for various soft-thresholding powers. **C** The heatmap identified groups of correlated eigengenes termed meta-modules. **D** TOM cluster dendrogram of WGCNA: Branches with different colors corresponding to different modules. Dynamic Tree Cut represents the original module, while Merged Dynamic represents the final module. **E** Heatmap of the correlation between gene modules and the immunity cluster of ovarian cancer. The purple module was the most significant module with immunity. **F** Scatter plot of module eigengenes in the purple module

In WGCNA analysis, we identified 13 co-expression modules and analyzed their association with the immune-related cluster from ssGSEA. Based on the lncRNAs, a co-expression network was established by R package “WGCNA”, which could reveal the modules and genes that were significantly associated with the immunity cluster (Fig. 2A). In this study, β = 5 was the best choice for soft thresholds to construct a scale-free network (Fig. 2B). We next visualized the gene network with the meta-modules (Fig. 2C). After adjusting the parameters of WGCNA, we classified the DEGs into 13 modules (Fig. 2D). The results indicated that purple module was the most correlated module of immunity-cluster (*r* = 0.69, *P* = 7e-34, Fig. 2E). There were 141 genes in the purple module (Table S1). In the module-trait analysis, 8 genes with GS value > 0.3 and MM value > 0.8 were defined as hub genes: CCDC69, CLMP, FAM110B, FAM129A, GUCY1B3, PALLD, PLEKHO1, and STY11. Afterward, we defined genes in the purple module as stemness-related hub genes (Fig. 2F). These results suggested that the genes in the purple module was significantly related to the stemness of OC cells.

### Function and DEGs of lncRNA related genes

**Fig. 3:**
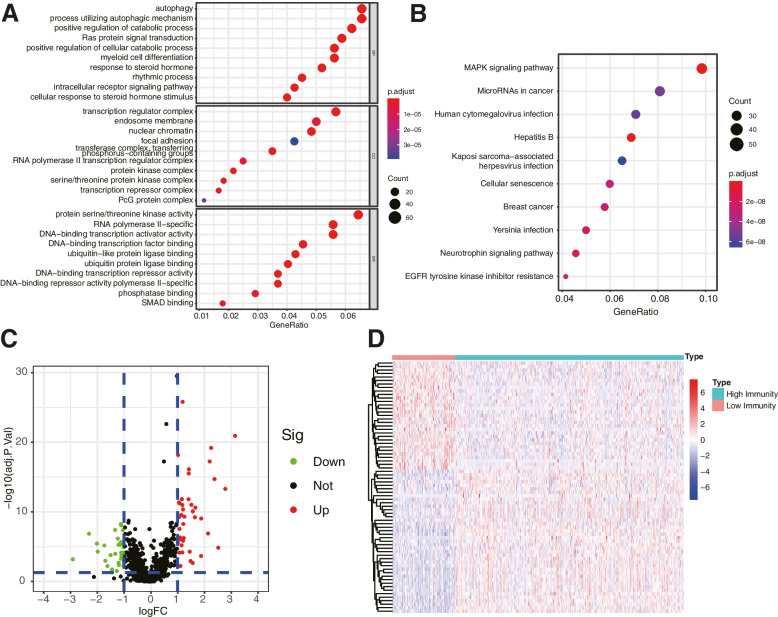
Enrichment analysis and differentially expressed genes of immune-related lncRNAs. **A** GO enrichment analysis and **B** KEGG pathway enrichment analysis was performed. **C** Volcano plot of DEGs. **D** Heatmap of the DEGs

We next conducted the correlation analysis for the key lncRNAs resulted from the WGCNA. Finally, 1269 genes were identified. Functional annotation analyses of the selected genes were then performed. GO enrichment showed that the lncRNA related genes were mainly involved in “autophagy”, “process utilizing autophagic mechanism”, “transcription regulator complex”, “focal adhesion”, and “protein serine/threonine kinase activity” (Fig. 3A). KEGG pathway enrichment analysis showed that the target genes were mainly involved in “MAPK signaling pathway”, “MicroRNAs in cancer”, and “Human cytomegalovirus infection” (Fig. 3B). Meanwhile, we compared the DEGs between normal and cancer tissues of the related genes. A total of 72 DEGs, were identified including 31 down-regulated genes and 41 up-regulated genes (Fig. 3C). The heatmap of DEGs were shown in Fig. 3D. These outcomes indicated that the key genes were functional in the progression of OC cells.

### Consensus cluster analysis for selected key gene expression profiles

**Fig. 4:**
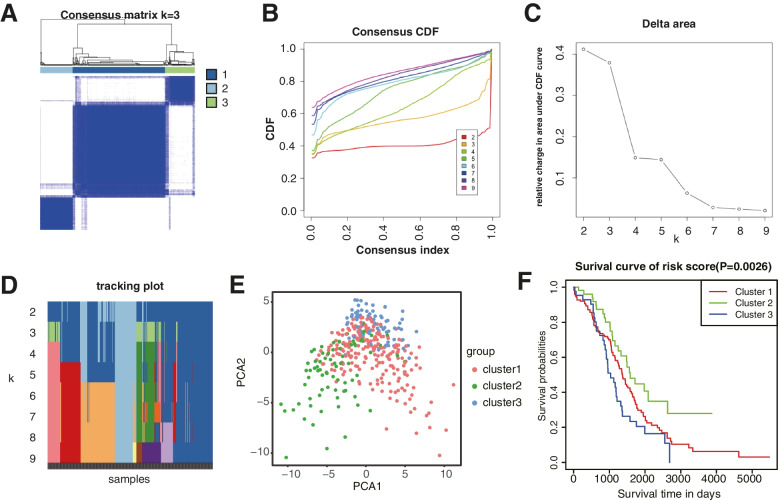
Identification of the molecular subtypes of the OC patients using the DEGs associated with prognosis. **A** The OC patients were stratified into 3 clusters based on the consensus clustering matrix (k = 3). **B-D** Consensus clustering model with cumulative distribution function (CDF) by k from 2 to 9. **E** The results of PCA analysis among the three subtypes. **F** Survival curves of patients in the three clusters

Then, we performed the consensus clustering analysis to investigate the relationship between these prognostic genes and OC subtypes. According to the CDF value, we classified the 379 OC patients into three clusters (k = 3, Fig. 4A–D). Cluster 1 (*n* = 226), cluster 2 (*n* = 80), and cluster 3 (*n* = 73) were generated from a total of 379 patients. We used principal component analysis (PCA) to display differences in gene expression levels among the three subgroups (Fig. 4E). We also found that the patients from cluster 2 tended to survive longer than the patients from cluster 1 and cluster 3 (Fig. 4F), implying a significant prognostic value of these DEGs.

### Establishment and validation of the risk signature based on lncRNA expression

**Fig. 5:**
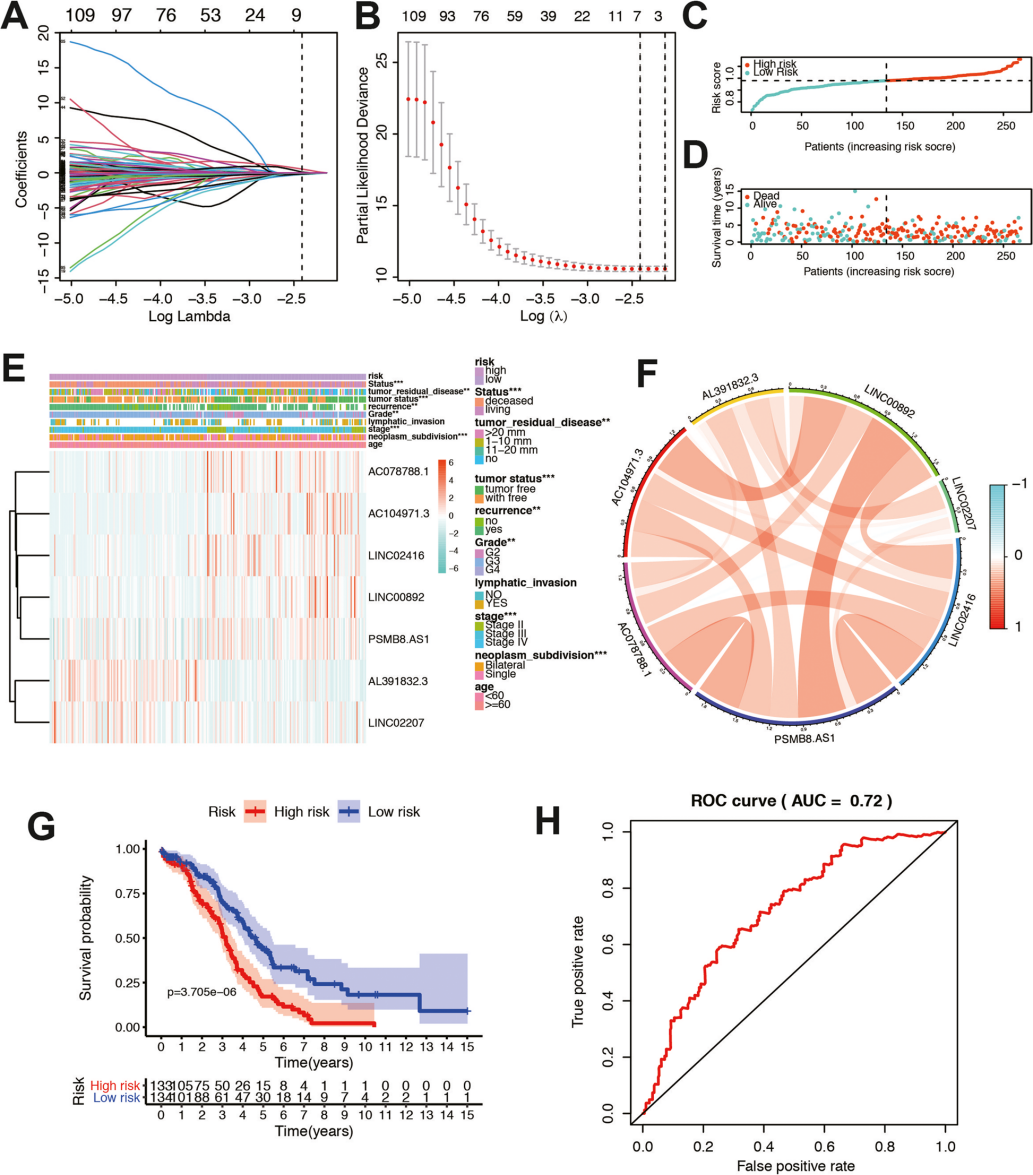
Construction of the immune-related lncRNA risk model in TCGA cohort. **A** LASSO coefficient profiles of the common genes. **B** Cross-validation for tuning parameter screening in the LASSO regression model. **C, D** Distribution of risk score, survival time and survival status. **E** Heatmap of the 7 lncRNA expression pattern in clinicopathologic characteristics and risk score in the TCGA database. **F** Correlation between the 7 lncRNAs. **G** Survival analysis for different risk groups in the combined TCGA-OC patients. **H** Time-dependent ROC curve analysis of the risk model

We then constructed a risk model by the lncRNA resulting from the purple module. First, we selected these genes to conduct an additional LASSO regression analysis on 136 lncRNAs (Fig. 5A-B). Table 1 listed the genes and coefficients used to calculate each subject’s risk score. The formula was as follows: Risk score = (AL391832.3*0.547455232)-(AC078788.1*0.219104082)- (AC104971.3*0.240874313) –(LINC00892*0.060063563) + (LINC02207*0.115620759)- (LINC02416*0.160019691) –(PSMB8.AS1*0.015774278). The risk scores of OC patients in TCGA were evaluated, and all patients were divided into high-risk group and low-risk group according to the median risk score (Fig. 5C). There was no doubt that the mortality rate in the high-risk group was considerably higher than that in the low-risk group (Fig. 5D). Differential-expression levels of the 7 lncRNA and clinicopathological features in the high- and low-risk groups are shown in heatmaps (Fig. 5E). The results showed that living status, tumor residual disease, tumor status, recurrence, grade, stage, and neoplasm subdivision were differentially distributed in the two risk groups. The correlation analyses were also performed to check the expression correlation between the hub genes. (Fig. 5F). To evaluate the role of the 7-lncRNA signature in OC, we drew K-M curves for the high- and low-risk groups of the TCGA-OC cohort (Fig. 5G). These two subgroups significantly differed in OS (*P* < 0.01). Thereafter, we used a time-dependent ROC curve to predict the efficacy of the risk signature. The area under the ROC curve (AUC) of the prediction model was 0.72 of the OS (Fig. 5H). The contents of seven lncRNAs in different immunity groups were also compared. As shown in supplementary Fig. S1, the expressions of seven lncRNAs were all elevated in immunity_H group. We also validated the function of PSMB8-AS1 in SKOV3 cell line in vitro and in vivo*.* As shown in supplementary Fig. S2, the metastatic capability significantly decreased after knocking down PSMB8-AS1. These results suggested that the 7 lncRNAs play essential roles in the progression of ovarian cancer.

**Table 1:** Seven immune cluster associated genes and corresponding coefficient value

Immune cluster associated gene	Coefficient
AC078788.1	-0.2191041
AC104971.3	-0.2408743
AL391832.3	0.54745523
LINC00892	-0.0600636
LINC02207	0.11562076
LINC02416	-0.1600197
PSMB8.AS1	-0.0157743
Risk score	Low: < 0.97
	High: ≥ 0.97

### Functional analysis of the risk score model

**Fig. 6:**
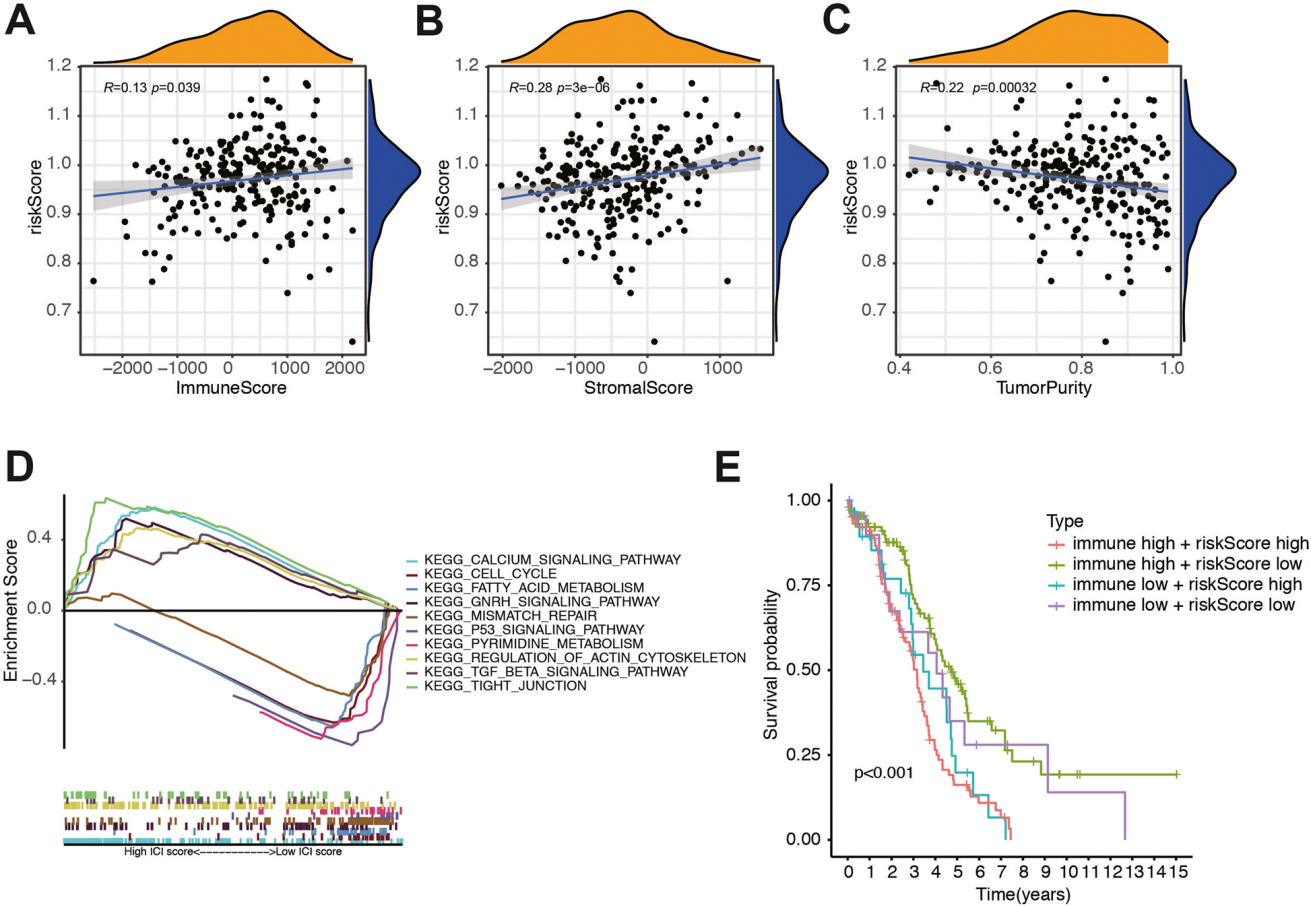
The association between tumor microenvironment and risk score. Discrepancy of **A** Immune Score, **B** Stromal Score and **C** Tumor Purity in two groups. **D** Functional enrichment analysis between low- and high-risk groups of top five signaling pathways in the high- and low-risk subgroup. **E** Survival analysis for four groups stratified by combining the immune signature and the risk score characteristic in the TCGA-OC cohort

We further investigated the correlation between the risk score and ESTIMATE related score including immune score, stromal score, and estimate score. We found a low positive relationship between Immune Score, Stromal Score and risk score with *r* = 0.13 and 0.28, respectively (both* p* < 0.01, Fig. 6A-B), which pointed out that stromal and immune cell was higher in the high risk group. However, the relationship between Tumor Purity and risk score was negatively correlated (Fig. 6C). These results indicated that patients with an unfavorable prognosis in the high risk group associated with the variation in tumor immune microenvironment of OC. To clarify the important pathway of signature enrichment related to the risk signature, we conducted GSEA. Finally, 55 enrichment pathways with significant variations between low and high-risk groups were identified at the criteria of FDR < 0.25, *P*-value < 0.05. The top five signaling pathways in the high-risk group were calcium signaling pathway, cell cycle, fatty acid metabolism, GnRH signaling pathway, and mismatch repair. On the other hand, the top five signaling pathways in the low-risk group were P53 signaling pathway, pyrimidine metabolism, regulation pf actin cytoskeleton, TGF-β signaling pathway, and tight junction (Fig. 6D). Furthermore, we stratified the patients into four subgroups according to the immune score and risk score. The result indicated that patients with high immune score and low risk score had the most favorable prognosis. However, patients with high immune score and high risk score had the worst prognosis (Fig. 6E). These results illustrated the relationship between ESTIMATE score and risk score was significant, and the potential function of the risk signature was also meaningful.

### Construction and validation of the prognostic-nomogram model

**Fig. 7:**
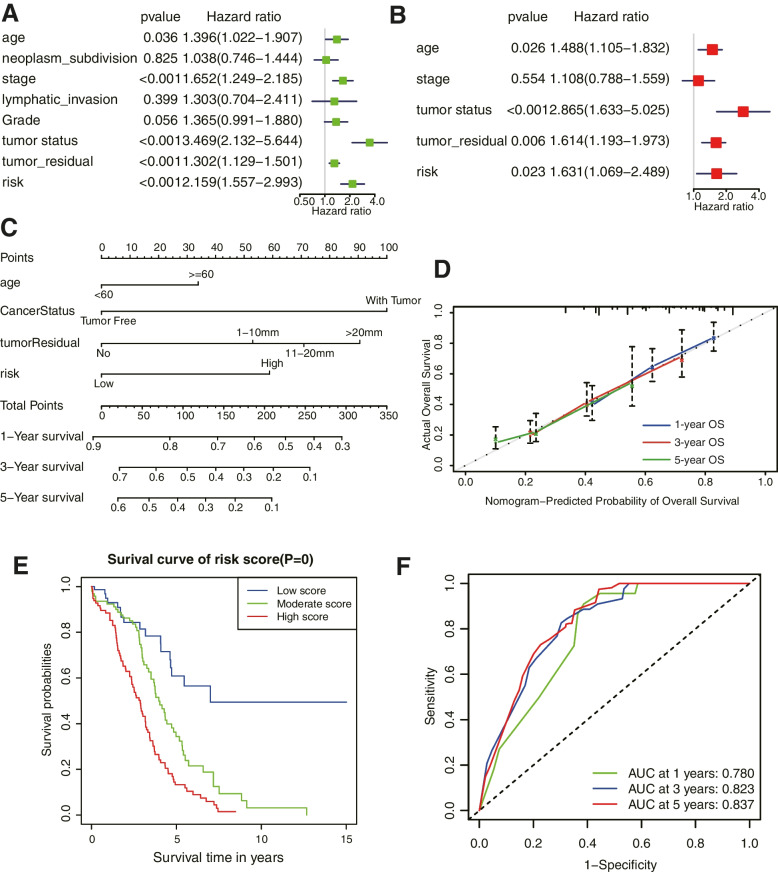
Construction and validation of the nomogram based on immune-related lncRNA signature and clinicopathological features. **A-B** Univariate and multivariate Cox regression analyses in the TCGA cohort. **C** The nomogram was established using age, tumor status, tumor residual, and the risk signature in the TCGA-OC cohort. **D** Calibration diagram of the nomogram for predicting the probability of OS at 1, 3, and 5-years. **E** Survival curve of patients in low, moderate, and high score groups. **F** Prediction of the nomogram indicated by AUC based on clinical traits and risk score

Next, we performed univariate and multivariate Cox regression analyses in the TCGA-OC patients to assess the independent prognostic value of the lncRNA related risk signature. We observed that in univariate analysis, age, stage, tumor status, tumor residual, and risk score were significantly correlated with prognosis (Fig. 7A). Furthermore, multivariate analysis indicated that age, tumor status, tumor residual, and risk score were independent prognostic factors in the TCGA-OC patients (Fig. 7B; both *P* < 0.05). A nomogram model based on four independent risk factors was established to evaluate the prognostic significance of the risk signature in OC patients (Fig. 7C). The corresponding score of each variable is shown in Table 2. The calibration curves revealed a favorable consistency between expected and observed survival rates (Fig. 7D). Then patients with OC were divided into three subgroups evenly according to the total points from the nomogram namely low-, moderate-, and high-score group. The overall survival curve of the three groups was shown in Fig. 7E. The results showed that patients with high scores had the worst prognosis. What’s more, the ROC showed that nomogram could accurately predict the survival outcome of patients, and the AUC values of 1, 3 and 5 years were 0.780, 0.823 and 0.837 respectively (Fig. 7F). Taken together, the results described above suggested that the nomogram model had good reliability in predicting OS in OC patients.

**Table 2:** Corresponding risk score for each variable and total score

Variables	Category	Score
Age	< 60	0
	≥ 60	33
Cancer status	Tumor free	0
	With tumor	100
Tumor residual	No	0
	1-10 mm	52.5
	11-20 mm	70
	> 20 mm	90
Risk signature	Low	0
	High	57.5
Total score	Low risk	0–95
	Moderate risk	100–215
	High risk	≥ 225

### Validation of the lncRNA-related risk signature in GEO database

**Fig. 8:**
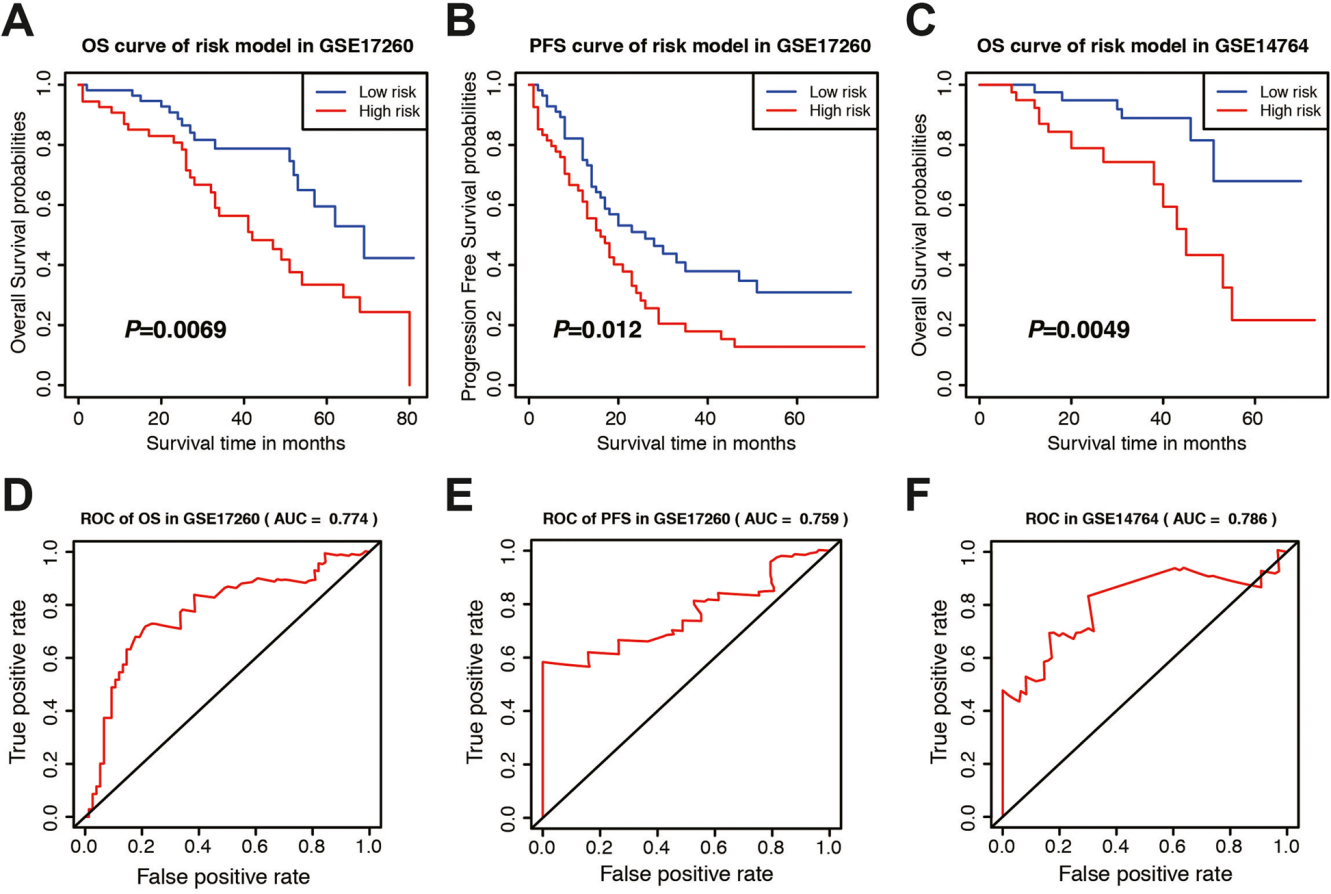
Verification of the immune-related signature in two independent cohorts. **A** OS plot of the lncRNA-related signature in the GSE17260. **B** PFS plot of the signature in the GSE17260. **C** OS plot of the signature in the GSE14764. **D** Time-dependent ROC curve of the lncRNA-related signature in GSE17260 of OS. **E** Time-dependent ROC curve of the signature in GSE17260 of PFS. **F** Time-dependent ROC curve of the signature in GSE14764 of OS

To assess the predictive value of the risk model, we used the risk score algorithm in the GSE datasets. The results in the validation cohort revealed that OC patients in the high-risk group had worse OS and PFS rates in GSE17260 (Fig. 8A-B), and OS in GSE14764 (Fig. 8C) than those in the low-risk group. The AUCs for survival were 0.774, 0.759, and 0.786, respectively (Fig. 8D-F). These findings suggested that the 7-lncRNA risk model could accurately predict the prognosis of patients with OC.

### Chemotherapeutic drug sensitivity analysis and validation

**Fig. 9:**
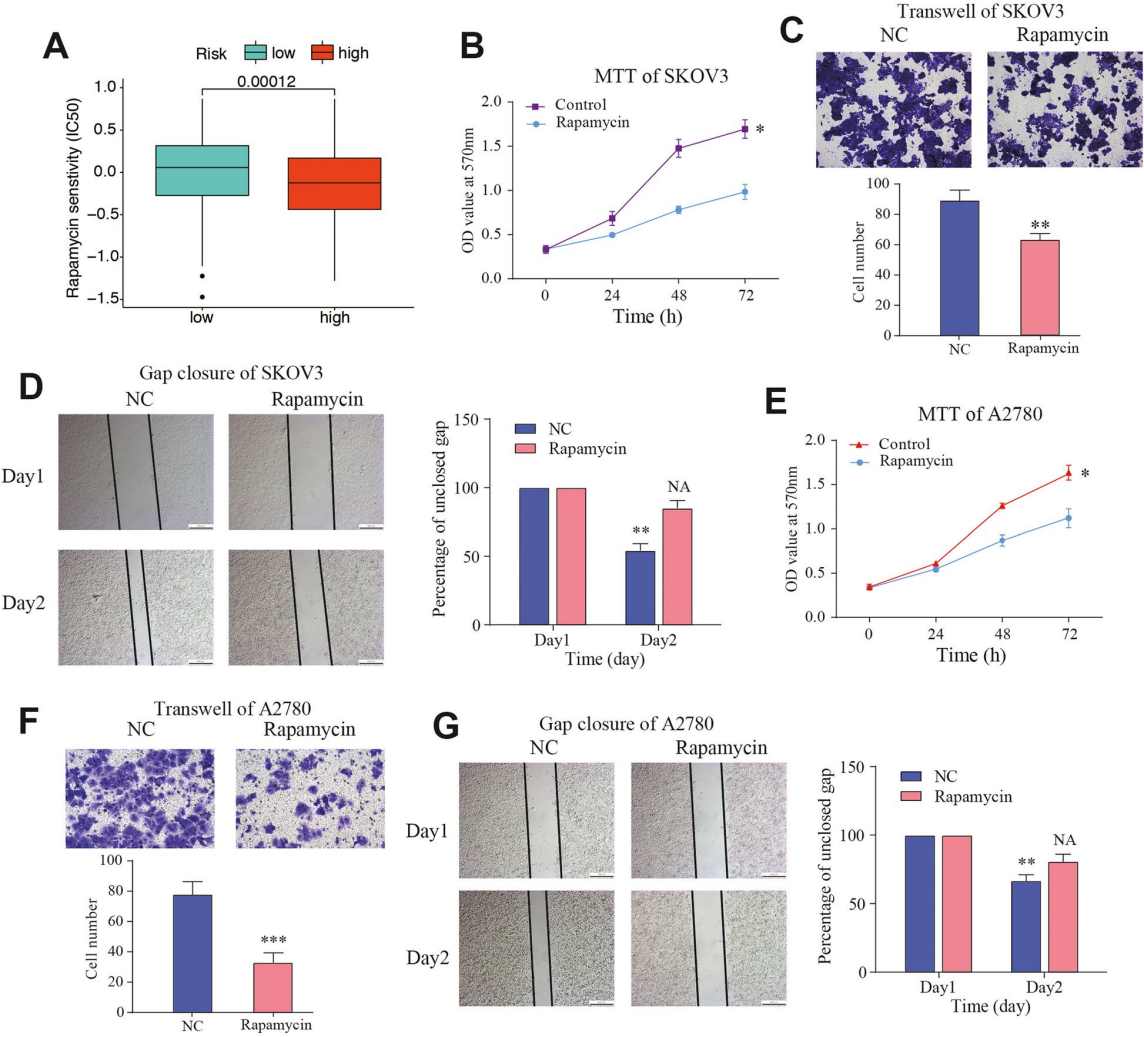
In vitro validation of rapamycin in OC cell lines. **A** Differences in drug sensitivity of rapamycin between high- and low-risk groups. **B** MTT assay, **C** transwell assay, and **D** gap closure analysis were performed in SKOV3 cell line after treated with rapamycin at the concentration of 20um. **E** MTT assay, **F** transwell assay, and **G** gap closure analysis were also performed in A2780 cell line after treated with rapamycin at the concentration of 20um. NC, Normal Control

To observe the differences in drug sensitivity of commonly used chemotherapeutic agents between the different risk groups, drug selection was used. “pRRophic” package is a method used to predict sensitivity of some kinds of chemotherapy drugs. By using the “pRRophetic” package for drug sensitivity analysis, we observed that patients in the high-risk group were more sensitive to rapamycin (Fig. 9A). SKOV3 is derived from ascites isolated cells from patients with ovarian cancer. It has resistance to some chemotherapy drugs including cisplatin and adriamycin. A2780 is similar with SKOV3 cell line. Therefore, we further validated the function of rapamycin by in vivo experiments with SKOV3 and A2780 cell lines. Transwell and wound healing experiments indicated that rapamycin inhibited invasion and metastasis in OC cell lines (Fig. 9B-C).

## Discussion

Ovarian cancer (OC) has high mortality rates because the early symptoms are uneasily detectable, and in most cases, cancer has already advanced to late stages when diagnosed. The high-grade serous subtype contributes to the majority of OC deaths, mainly as a result of the advanced stage of patients upon initial diagnosis and the high likelihood of relapse after chemotherapy. Therefore, there is an urgent need to develop reliable tumor markers and explore accurate prognostic strategies for the treatment of OC [19, 20]. Accumulating evidence suggests that lncRNAs play important roles in the occurrence and development of tumors. LncRNAs participate in a range of biological events and are known to regulate tumorigenic processes. For example, inhibition of HOXD-AS1 reduced OC cell migration, invasion, and epithelial-mesenchymal transition (EMT) in OC cells in vitro by preventing HOXD-AS1 directly binding to miR-186-5p, and resulting in down-regulating of PIK3R3 [21]. Overexpression of lncRNA CTBP1-DT could competitively bind to miR-188-5p to protect MAP3K3 from degradation, which could promote malignant biological behaviors of HGSOC (high-grade serous ovarian cancer) cells [22]. To accurately predict the clinical outcomes or chemotherapy resistance of OC patients and improve their long-term survival, the development of novel molecular biomarkers for early OC detection is a high priority [23].

In order to verify the importance of immune-related lncRNAs in ovarian cancer progress, lncRNA- related prognostic and diagnostic model were developed. In this study, we used ssGSEA to identify immune-related subtypes and estimate the enrichment degree of 29 gene sets in each sample of TCGA-OC. WGCNA was conducted to reveal the key lncRNAs that played important roles in this immune cluster. Then, a prognostic model integrating lncRNAs were constructed through the LASSO regression analysis methods. The expressional level of the seven lncRNAs, which were used to construct the risk model, were also compared between low and high immunity clusters. This results indicated that population in high immunity cluster is mostly affected by lncRNA regulation. Furthermore, we successfully divided OC samples into two groups, high- and low-risk groups based on median risk score. Low-risk group had a better prognosis for OS and had a higher immune infiltration level than high-risk group. We then used OC cell line to verify the predictive value of the risk score and found that it can predict the prognosis and chemotherapy sensitivity of rapamycin drugs. Finally, we performed a cell migration and invasion assay and found that the ability to metastasize cells was significantly decreased after dealing with rapamycin. This explained that the risk score predicting prognosis may be due to invasion and metastasis of predicted drug. The prediction efficiency of our model can be verified through gene expression matrixes of other datasets in different platform, which confirms the reliability and feasibility of our research.

GO and KEGG analyses indicated that these lncRNAs are involved in autophagy, focal adhesion, Ras protein signal transduction, and positive catabolic process. Autophagy-related lncRNAs has been reported to be potential as an independent prognostic indicator in endometrial cancer and ovarian cancer [24, 25]. GAS8-AS1 inhibited OC progression by activating autophagy via binding with Beclin1, which could be reversed by rapamycin. Autophagy-related lncRNA might be a potential therapeutic target for OC clinical treatment [26]. Focal adhesion is an essential function and play an important part in the progression of cancer. For example, LRRC15 expression leads to inhibition of anoikis-induced cell death and promotes adhesion and invasion through matrices that mimic omentum [27]. Oncogenic RAS mutations drive cancers at many sites. Disruption of K-RAS cluster formation requires the N terminus of DIRAS3 and interaction of both DIRAS3 and K-RAS with the plasma membrane. Interaction of DIRAS3 with both K-RAS and H-RAS suggests a strategy for inhibiting oncogenic RAS function [28]. Although metabolism and some subclasses of nutrition may be associated to EOC risk, lipid metabolism of LPA (lysophosphatidic acid) and AA (arachidonic acid) emerges as an important signaling network in epithelial ovarian carcinomas (EOC) [29]. As an mTOR inhibitor, rapamycin is closely related with lncRNAs. Increased expression of lncRNA CASC9 promoted tumor progression by suppressing autophagy-mediated cell apoptosis via the AKT/mTOR pathway [30]. LINC01554 could promote the ubiquitin-mediated degradation of PKM2 and inhibited Akt/mTOR signaling pathway to abolish aerobic glycolysis in tumor cells [31].

Gene markers are widely used in modern clinical diagnosis. Collectively, our results suggest that the seven lncRNAs may serve as biomarkers to predict the survival and act as key molecules to reveal potential mechanism for patients with OC. LINC00892 involved in molecular subtype and risk model may be useful in improving the prognostic prediction of bladder cancer patients with different clinical situations and may help to find a useful target for tumor therapy [32]. It was also associated with immune cell infiltration and immune checkpoint inhibitors immunotherapy-related biomarkers such as mismatch repair (MMR) genes, tumor mutation burden (TMB) and immune checkpoint genes [33]. However, the specific function of LINC00892 in OC is still unclear. We proved its function and found that the expression of LINC00892 is high in immunity_H cluster. The further functional pathway needs to be explored. It was reported that PSMB8.AS1 is enriched in immune response processes, which increased CD8 T-cell tumor infiltration and trans-regulation of genes in immune-related pathways, suggesting that an epigenetically mediated immune response is a predictor of recurrence and, possibly, treatment response for high-grade serous (HGS) EOC [34]. LINC02207 was also identified as a predictive marker with significant prognostic value in ovarian carcinoma [35]. Another study found that lncRNA HOXA11.AS knockdown increased the expression of autophagy-related proteins and improved cisplatin sensitivity, decreased ovarian cancer cell proliferation, and promoted cell apoptosis [36]. As an epithelial-mesenchymal transition (EMT) related lncRNA [37], researchers found that PMSB8.AS1 promoted pancreatic cancer (PC) progression via STAT1 by sponging miR-382-3p involving regulation PD-L1 [38]. Knockdown of PMSB8.AS1 could also suppress EMT of PC cells. The downregulation of PSMB8.AS1 repressed cell viability and EMT of colorectal cancer while promoting its apoptosis [39]. All these studies illustrated that lncRNAs functioned as key molecules in the pathogenesis and progression of ovarian cancer.

There were some other studies concentrating on different features of biological process and the comprehensive analyses of functional related genes for OC [40, 41], especially for lncRNAs. For example, one study identified and validated risk model based on five immune-related lncRNAs is an independent prognostic factor for OC patients. The two risk groups were confirmed to be sensitive to several chemotherapeutic agents and patients in the low-risk group were more sensitive to immunotherapy [42]. Another study identified five prognostic genes associated with immune infiltration of OC. Some significant variations of copy number on gene loci were found between two risk groups and it showed that patients with fine chemo-sensitivity has lower risk score than patient with poor chemo-sensitivity [43]. A 5-lncRNA signature of prognostic value was established for survival prediction, and also constructed ceRNA networks for exploration of potentially more selective drugs for OC [44]. All these studies revealed that lncRNAs are potential biomarkers for the prediction and prognosis of patients with OC. Furthermore, a nomogram integrating the risk model and clinicopathological features are established. The nomogram model is considered to be an evidence-based, accurate method for the assessment of treatment and prognosis, and has been widely used in studies on a variety of OC study [45, 46]. A nomogram prediction model was successfully constructed on the base of independent risk factors determined through survival analyses. By incorporating independent risk factors into nomogram modeling to predict the survival rate, an AUC of 0.837 was achieved, indicating the excellent predictive ability of this method. The model can predict the survival rate of individual patients and is helpful for clinical treatment decision-making and design of clinical re- search programs.

According to the currently searchable literature, this is the first relatively comprehensive study to establish an immune cluster-related lncRNA prognostic model for patients with OC and develop prognostic-related line graphs. However, some limitations should also be noted in our study. First, it is a retrospective study, for some of the cohorts used, important clinical indicators including surgery type, time to recurrence and metastasis is not available due to the loss of patients and even if there are strict standards, information bias is likely to appear. Another flaw of this study is that due to the limited number of OC samples that can annotate lncRNA expression, more patients with homologous information were needed to incorporate into study and prove the credibility of our study. Last but not least, the sensitivity of rapamycin should be verified in human samples to prove its antitumor effect.

## Conclusion

In conclusion, this study shows that a signature consisting of 7 lncRNAs that has potential clinical value for the early diagnosis and prognostic monitoring of OC was identified for prognosis prediction in patients with OC, where a higher risk score indicates poorer prognosis. Further research of underlying mechanisms based on these lncRNAs may facilitate and provide some landscape for individualized treatment of OC.

## Supplementary Information

**Additional file 1: Table S1.**

**Additional file 2: Fig. S1.** Expression levels of 7 selected lncRNAs in different immunity clusters.

**Additional file 3: Fig. S2.** Functional analysis of PSMB8-AS1 in SKOV3 cell line in vitro and in vivo. A Knockdown efficiency of PSMB8-AS1 in SKOV3 cells in protein level. B Gap closure for PSMB8-AS1 in SKOV3 cell line. C In vivo experiments used to verify the function of PSMB8-AS1. NC, Negative Control.

## Data Availability

The datasets analyzed during the current study are available in the [TCGA] repository, [https://portal.gdc.cancer.gov/].

## Abbreviations

OC: Ovarian cancer
TCGA: The Cancer Genome Atlas
DEGs: Differentially expressed genes
WGCNA: Weighted gene co-expression network analysis
ssGSEA: Single sample GSEA
LASSO: Least absolute shrinkage and selection operator
GEO: Gene Expression Omnibus
immunity_L: Low immunity
immunity_H: High immunity
lncRNAs: Long noncoding RNAs
TME: Tumor microenvironment
ESTIMATE: Estimation of Stromal and Immune cells in Malignant Tumor tissues using Expression
TFs: Transcription factors
HLA: Human leukocyte antigen
MEs: Module eigengenes
GS: Gene significance
KEGG: Kyoto Encyclopedia of Genes and Genomes
GO: Gene Ontology
BP: Biological processes
MF: Molecular functions
CC: Cellular components
OS: Overall survival
ROC: Receiver operating characteristic
AUC: Area under the ROC curve
FBS: Fetal bovine serum
