# Supplementary figures and images for: Development and verification of a 7-lncRNA prognostic model based on tumor immunity for patients with ovarian cancer
Source: J Ovarian Res. 2023 Feb 4;16:31. doi: 10.1186/s13048-023-01099-0 (PMC9898952; doi:10.1186/s13048-023-01099-0)

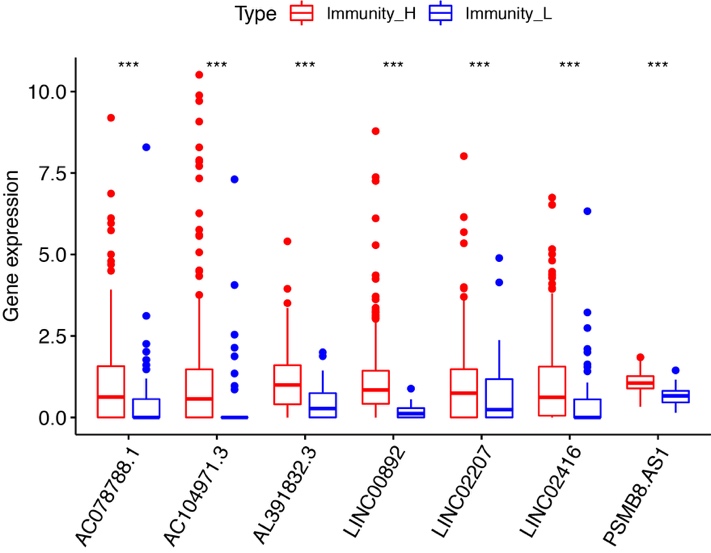


**Fig. S1**: Expression levels of 7 selected lncRNAs in different immunity clusters.

Supplement: Supplementary file 2 — Additional file 2: Fig. S1. Expression levels of 7 selected lncRNAs in different immunity clusters. [file 13048_2023_1099_MOESM2_ESM.docx]
