# Supplementary material for: Development and verification of a 7-lncRNA prognostic model based on tumor immunity for patients with ovarian cancer
Source: J Ovarian Res. 2023 Feb 4;16:31. doi: 10.1186/s13048-023-01099-0 (PMC9898952; doi:10.1186/s13048-023-01099-0)
Supplement: Supplementary file 3 — Additional file 3: Fig. S2. Functional analysis of PSMB8-AS1 in SKOV3 cell line in vitro and in vivo. A Knockdown efficiency of PSMB8-AS1 in SKOV3 cells in protein level. B Gap closure for PSMB8-AS1 in SKOV3 cell line. C In vivo experiments used to verify the function of PSMB8-AS1. NC, Negative Control. [file 13048_2023_1099_MOESM3_ESM.docx]

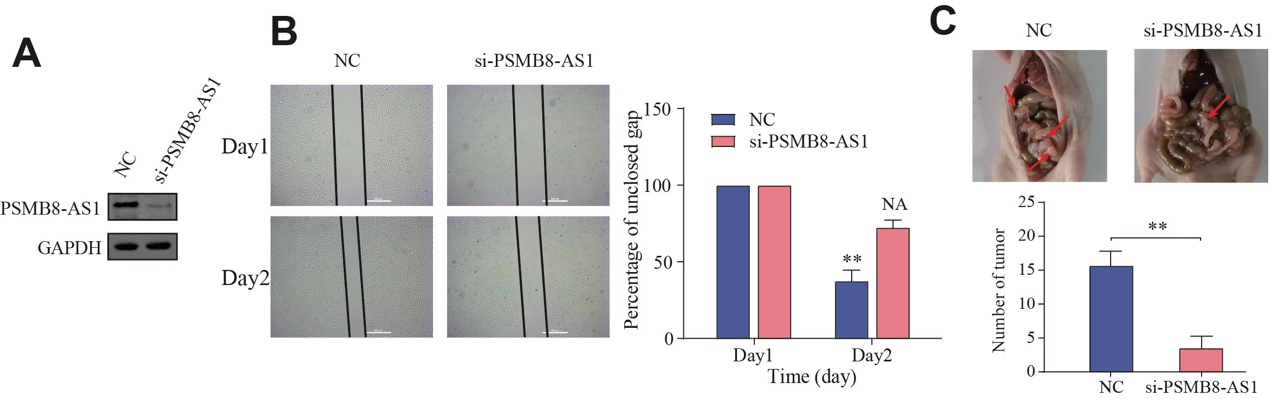


**Fig. S2**: Functional analysis of PSMB8-AS1 in SKOV3 cell line *in vitro* and *in vivo*. **A** Knockdown efficiency of PSMB8-AS1 in SKOV3 cells in protein level. **B** Gap closure for PSMB8-AS1 in SKOV3 cell line. **C** *In vivo* experiments used to verify the function of PSMB8-AS1. NC, Negative Control.
